# Can Countermovement Jump Neuromuscular Performance Qualities Differentiate Maximal Horizontal Deceleration Ability in Team Sport Athletes?

**DOI:** 10.3390/sports8060076

**Published:** 2020-05-27

**Authors:** Damian J. Harper, Daniel D. Cohen, Christopher Carling, John Kiely

**Affiliations:** 1 Institute of Coaching and Performance, School of Sport and Health Sciences, University of Central Lancashire, Preston PR1 2HE, UK; JKiely@uclan.ac.uk; 2 Masira Research Institute, Faculty of Health Sciences, University of Santander (UDES), Bucaramanga 680005, Colombia; danielcohen1971@gmail.com; 3 Mindeporte (Colombian Ministry of Sport) High Performance Centre, Bogota 111071, Colombia; 4 Centre for Elite Performance, French Football Federation, 75015 Paris, France; ccarling@fff.fr

**Keywords:** eccentric, concentric, force, impulse, velocity

## Abstract

This investigation aimed to determine the countermovement jump (CMJ) neuromuscular performance (NMP) qualities that differentiate between athletes with high or low horizontal deceleration ability. Twenty-seven male university team sport athletes performed a CMJ on vertical axis force plates and a maximal horizontal deceleration following a 20 m maximal horizontal sprint acceleration. The instantaneous velocity throughout the maximal horizontal deceleration test was measured using a radar device. The deceleration ability was evaluated using the average deceleration (HDEC, m·s^−2^) and change in momentum—referred to as the horizontal braking impulse (HBI, N·s·kg^−1^). Participants were dichotomised into high and low HDEC and HBI according to a median-split analysis, and CMJ variables calculated for the overall eccentric, eccentric-deceleration and concentric phases. When horizontal deceleration ability was defined by HDEC, the CMJ concentric (effect size (ES) = 0.95) and eccentric (ES = 0.72) peak forces were the variables with the largest difference between groups. However, when defined using HBI, the largest difference was the concentric (ES = 1.15) and eccentric (ES = −1.00) peak velocities. Only the concentric mean power was significantly different between the high and low groups for both HDEC (ES = 0.85) and HBI (ES = 0.96). These findings show that specific eccentric and concentric NMP qualities may underpin the horizontal deceleration abilities characterised by HDEC and HBI. Specific NMP training interventions may be beneficial to target improvements in either of these measures of horizontal deceleration abilities.

## 1. Introduction

High-intensity accelerations and decelerations are fundamental components of powerful movement actions in team sports and are integral to successful performance outcomes [1]. When compared to accelerations, high-intensity decelerations exhibit a greater rate of velocity change and subsequently occur more frequently in many team sports [2]. As an example, rapid decelerations are a pre-requisite for the many fast change of direction (COD) movements performed during team sport activities and can serve to enhance COD performance [3]. Additionally, high-intensity decelerations impose greater mechanical loads than comparable accelerations and demand that players withstand high eccentric braking forces [4,5]. Consequently, high-intensity decelerations are a component of the external load that disproportionately drives neuromuscular fatigue and thereby simultaneously escalating the risk of tissue damage [6].

However, despite this apparent importance, only a small number of studies have attempted to profile players’ maximal horizontal deceleration abilities using a horizontal sprint acceleration-to-deceleration task that requires players to fully stop before a specific boundary [7] or after covering a pre-determined acceleration distance [8,9]. All these previous studies used deceleration distance-to-stop or deceleration time-to-stop to calculate the horizontal deceleration abilities. As with maximal sprint acceleration profiling, radar and laser devices have been recommended as a means to profile the maximal horizontal deceleration abilities in substantially greater detail than previously possible [10]. Furthermore, deceleration demands are elevated in players with higher body mass as, for comparable changes in velocity, they inevitably achieve higher horizontal sprint momentums before commencing deceleration. Therefore, it also seems important to take into consideration how well a player changes their momentum.

Currently, the key underlying neuromuscular performance (NMP) qualities that potentially differentiate between athletes of varying deceleration abilities remain largely unexplored. It has been suggested that deceleration ability is underpinned by four major NMP qualities, namely dynamic balance, eccentric strength, reactive strength and power [11]. However, only a limited number of empirical studies have investigated the importance of these NMP qualities regarding horizontal deceleration abilities [3,7,8,9]. Interestingly though, these investigations all concluded that a greater eccentric strength of the quadriceps or hamstrings was beneficially associated with various measures of horizontal deceleration ability (i.e., negative change in velocity, deceleration gradient, time to stop or distance to stop). Additionally, in the study by Harper et al. [8], concentric peak torque measured at higher knee joint angular velocities had the strongest association with both the deceleration distance and time to stop, suggesting that this NMP quality is also an important determinant of horizontal deceleration ability.

The countermovement jump (CMJ) is commonly used within athletic performance settings to assess lower limb dynamic NMP capabilities [12]. When the CMJ is measured using force plates, deeper insights into an athlete’s NMP can be obtained by examining kinetic variables derived from the force–time curve captured during the eccentric (descent) and concentric (ascent) phases of the jump [13]. The eccentric phase can be further divided into two sub-phases: unloading or eccentric acceleration, and eccentric deceleration [14], also referred to as the eccentric-braking phase [13]. Eccentric performance is principally evaluated within the eccentric-deceleration phase, during which, the ability to decelerate the centre of mass (COM) immediately before the concentric phase is assessed [14]. Variables typically measured within this phase include the eccentric-deceleration rate of force development (RFD), eccentric-deceleration impulse, eccentric peak force and eccentric peak power. Eccentric-deceleration RFD, eccentric peak force and eccentric peak power are responsive to strength, power and plyometric training [14,15,16], with values differing for eccentric-deceleration RFD by sport [17,18], strength level [15] and type of strength exercises used within the training programme [19]. Eccentric-deceleration impulse has been shown to discriminate between developmental level within a sport [20,21] and gender [22], but seems less sensitive than eccentric-deceleration RFD to detecting inter-limb asymmetries following return-to-sport [23] and short-term strength and power training [16]. It is also important to consider that eccentric variables significantly contribute to performance in the subsequent concentric phase [15,17,24,25]. For example, increased force and COM velocity in eccentric phases have been associated with increased neural contractile capacity, leading to less fascicle lengthening and the enhanced contribution of tendon tissue to the force output in the concentric phase [15]. Consequently, there are a variety of eccentric variables in the CMJ that may reflect unique performance adaptations and movement capabilities; however, these have not been explored in the context of their association with maximal horizontal deceleration ability.

Nevertheless, it has also been suggested that athletes demonstrating superior eccentric force production capabilities during the CMJ, possess expanded repertoires of potential horizontal deceleration strategies [24]. Potentially, NMP qualities quantifiable during the different phases of the CMJ may provide insights into some of the critical qualities underpinning maximal horizontal deceleration ability. This information could be valuable in the design of training interventions that target the development of a team sport player’s maximal horizontal deceleration ability and to determine whether training is promoting the desired NMP adaptations that may lead to improvements in horizontal deceleration ability. Additionally, identifying indirect indices of horizontal deceleration ability in the CMJ, an assessment commonly employed in weekly monitoring within team sports, could provide valuable snapshots of neuromuscular status, specifically relating to NMP characteristics associated with the ability to produce and attenuate high eccentric forces during rapid decelerations [6].

Therefore, the purpose of this study was to evaluate whether athletes determined to have high or low horizontal deceleration ability displayed differences in CMJ eccentric and concentric NMP variables. Additionally, since the purpose of deceleration in team sports contexts is to decrease the body’s momentum (mass × velocity) [26], we aimed to determine whether CMJ NMP variables differ according to whether horizontal deceleration ability is quantified using a negative change in momentum (referred to as “horizontal braking impulse”) versus a negative change in velocity (referred to as “horizontal deceleration”). We hypothesized that both CMJ eccentric and concentric phase NMP variables would differ between athletes with a high versus low horizontal deceleration ability and that these associations would vary when this ability was defined by horizontal deceleration compared to horizontal braking impulse.

## 2. Methods

### 2.1. Participants

Twenty-seven male university sports athletes (age: 19.7 ± 1.7 years, height: 176 ± 10 cm, body mass: 73.0 ± 14.7 kg) who participated primarily in team sports (soccer, rugby league, rugby union) volunteered to participate. To be eligible for inclusion in the study, all participants had to take part in regular (three times per week) moderate to high-intensity exercise and be familiar with COD movements that involve high-intensity accelerations and decelerations. Participants were excluded from the study if they had suffered any kind of musculoskeletal injury that had prevented participation in sport or physical activity within the previous 3 months. All testing was conducted in December, which is mid-way through the University competitive sports season. The institutional ethics review committee at the University of Central Lancashire granted ethical approval in accordance with the recommendations of the Declaration of Helsinki. All participants received a clear written and verbal explanation of the study, including the benefits and risks of participation. Participants were also allowed to ask any questions prior to testing before providing voluntary, informed, written consent.

### 2.2. Experimental Design

A cross-sectional research design was used to investigate differences in CMJ NMP characteristics between athletes determined to have high and low horizontal deceleration ability. All experimental procedures took place over two weeks, in which participants were required to complete three testing sessions with at least 48 h recovery between them. Participants were asked to refrain from exercise in the 48 h before testing. In the first session, all participants had anthropometric measurements taken, completed a 20 m linear sprint and were familiarised with the protocols of the maximal horizontal deceleration test. In the second session, participants completed the maximal horizontal deceleration test. In the final session, participants completed CMJ testing. All testing was completed at the same time of the day (9:00 a.m. to 12:00 p.m.) on an indoor artificial sports surface. Before testing, all participants completed the same 15-min standardised warm-up that included forward and backward jogging, dynamic stretching and test-specific exercises (i.e., horizontal accelerations and decelerations, CMJ) following a progressive increase in intensity (70%, 80% and 100% perceived effort).

### 2.3. Testing Procedures

#### 2.3.1. Anthropometrics

Standing height was measured to the nearest cm using a stadiometer (Seca 217, Hamburg, Germany) and body mass was measured to the nearest 0.1 kg using electronic weighing scales (Seca, Hamburg, Germany).

#### 2.3.2. Maximal Horizontal Sprint Test

Sprint times were recorded over a 20 m distance using timing gates (Witty, Microgate, Bolzano, Italy) set to a height of 0.8 m [27]. Times were recorded to the nearest 0.01 s. Each sprint commenced from a stationary split stance position with the front foot positioned 30 cm behind the timing gate to prevent a false trigger. Participants were instructed to initiate their start with no backward step or “rocking motion” and to sprint as fast as possible. Each participant was allowed two trials with at least a 2 min recovery period. The best 20 m split was recorded as a “criterion” time for the maximal horizontal deceleration test.

#### 2.3.3. Maximal Horizontal Deceleration Test

**Figure 1:**
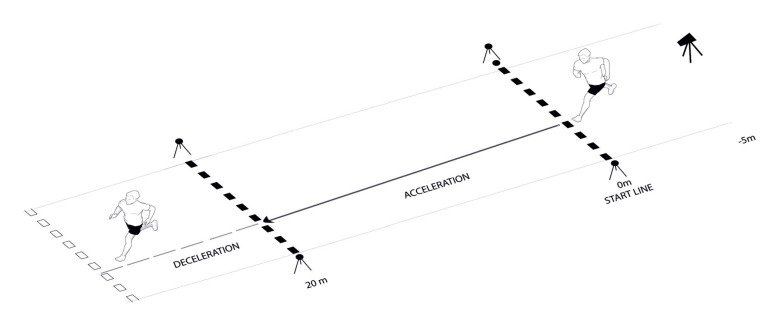
Acceleration–deceleration ability (ADA) test layout used to assess players’ maximal horizontal deceleration ability.

Maximal horizontal deceleration ability was assessed using a horizontal acceleration–deceleration ability (ADA) test [8]. Participants were instructed to use the same start protocol employed for the linear sprint test and to sprint maximally over 20 m before performing a maximal horizontal deceleration. Immediately following the deceleration, players backpedalled to the 20 m line to create a clear “stop” event and to signify the end of the deceleration phase (Figure 1). Any 20 m time that was 5% greater than the best 20 m split time achieved during the horizontal sprint test was considered an unsuccessful trial. The player was subsequently asked to repeat the test following at least a 3 min recovery period. Players were asked to perform a maximum of five trials, with the best two successful trials used for analysis [28].

Instantaneous horizontal velocity was measured throughout the maximal horizontal deceleration test using a radar device (Stalker ATS II, Applied Concepts, Inc., Dallas, TX, USA) sampling at 47 Hz. The radar device was mounted on a heavy-duty tripod and positioned 5 m behind the start line, which is within the 4.6 to 9.6 m distance recommended by the manufacturer for recording acceleration and braking run tests. The radar device was set to a height 1 m above the ground to approximately align with the participant’s COM.

#### 2.3.4. Radar Data Analyses

Raw instantaneous velocity–time data captured with the radar was manually processed using the Stalker ATS system software (Version 5.0, Applied Concepts, Inc., Dallas, TX, USA) following procedures outlined by Simperingham et al. [28] and then exported to Microsoft Excel (version 14.6.4, Microsoft, Redmond, DC, USA). Using processed data, the start of the deceleration phase was defined as the time point immediately following maximum velocity (V_max_). The end of the deceleration phase was defined as the lowest velocity (V_low_) following V_max_. The maximal horizontal deceleration ability was assessed using two methods: (1) average horizontal deceleration (HDEC) and (2) average horizontal braking impulse (HBI). Both of these variables were calculated from the average of the instantaneous HDEC and HBI data points captured from the start to the end of the deceleration phase. The instantaneous horizontal deceleration was calculated between each data point throughout the entire deceleration phase using the following equation:

(1) $$\mathrm{Deceleration}\;\left(m\cdot s^{-2}\right)=\frac{\left(v_{f}-v_{i}\right)}{\left(t_{f}-t_{i}\right)},$$

where *v* is the velocity, *t* is the time, *f* indicates the final velocity or time and *i* indicates the initial velocity or time.

The instantaneous HBI was calculated between each data point throughout the entire deceleration phase using the following equation to calculate the change in momentum:

(2) $$J\left(t\right)=M_{f}-M_{i}$$

where *J* is the impulse, *M_f_* is the final momentum and *M_i_* is the initial momentum.

The instantaneous momentum was calculated for each data point throughout the entire deceleration phase using the following equation:

(3) Momentum (t)=v×mass

The coefficient of variation (CV%) values calculated from the two best trials were 4.3% for HDEC and 3.7% for HBI, demonstrating excellent absolute reliability.

#### 2.3.5. Countermovement Jump (CMJ)

CMJs were performed with each foot positioned on a portable vertical axis force plate (35 × 35 cm, PASPORT force plate, PS-2141; PASCO Scientific, Roseville CA) that simultaneously sampled at a rate of 1000 Hz. To ensure the safety of participants during the CMJ landing phase, the force platforms were positioned within a heavy-duty foam surround. This portable dual force platform system has been shown to obtain valid measures of CMJ force–time variables in comparison to a laboratory ground-based force platform system [29]. Participants were instructed to perform a series of five CMJs interspersed with a 20 s recovery period. Before each jump participants were instructed to keep still and following a “3-2-1” countdown to jump for maximal height following a fast countermovement to the self-selected depth. All CMJs were performed with hands positioned on the hips. If hands were removed from the hips or knees flexed following takeoff, the jump was ruled invalid, and participants were asked to perform additional jump(s) at the end of the series.

#### 2.3.6. Force Platform Analyses

**Figure 2:**
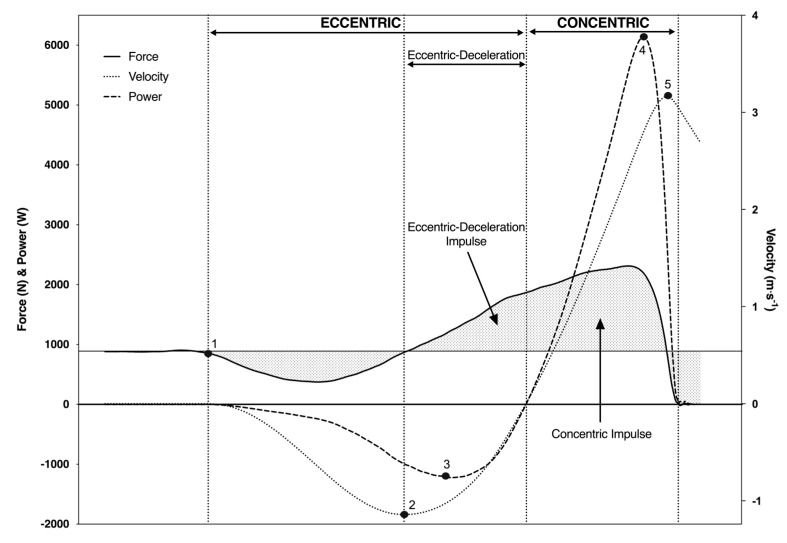
Force–, power– and velocity–time curves captured throughout each phase of the CMJ. Note: 1—start of movement, 2—eccentric peak velocity (start of eccentric-deceleration phase), 3—eccentric peak power, 4—concentric peak power, 5—concentric peak velocity.

CMJ force–time data were acquired and analysed using commercially available software (ForceDecks, Vald Performance Pty Ltd., Brisbane, Australia), which calculates a range of NMP variables that characterise performance during the jump. The definition and absolute reliability (coefficient of variation, CV%) for each CMJ NMP variable are reported in Table 1. All CMJ kinetic variables were divided by body mass to enable normalization amongst participants. Sample force–, power– and velocity–time curves during the eccentric and concentric phases of the CMJ are shown in Figure 2. Before each CMJ test being performed, the force-plates were zeroed using the manufacturer’s software. The start of the CMJ (movement onset) was defined using a 20 N offset from the measured body mass. Body mass was measured using the dual force plates over at least one second, in which the participant was asked to stand upright and as still as possible [13]. The CMJ takeoff was defined as the time point in which the vertical force dropped below a threshold of 20 N. The best and worst score for each CMJ variable was removed and the average of the remaining three scores was used for analysis.

### 2.4. Statistical Analysis

The mean ± SD was calculated for all CMJ variables. Participants were dichotomised into high- and low-horizontal-deceleration-ability groups based on a median split of the HDEC performance and separately split based on HBI performance. The differences in mean CMJ NMP variables between high- and low-deceleration-ability groups were examined using the independent samples *t*-test and Cohen’s *d*_s_ effect size with 90% confidence intervals, calculated using an online Microsoft Excel spreadsheet [30]. The magnitude of the effect size was interpreted using thresholds as suggested by Cohen [31]: 0.0 to 0.19—trivial; 0.20 to 0.49—small; 0.50 to 0.79—moderate; >0.80—large. The common language (CL) effect size was also calculated using the online Microsoft Excel spreadsheet to provide a percentage probability of a player from the high-deceleration-ability group having a greater measurement than someone from the low-deceleration-ability group [30]. Statistical significance was set at *p* < 0.05.

## 3. Results

Descriptive information showing differences between the high- and low-HDEC and -HBI groups are reported in Table 2. The high-deceleration-ability group reported significantly (*p* < 0.01) higher values for HDEC (−4.99 vs. −4.24 m·s^−2^, *d*_s_ = 2.21) and HBI (−8.43 vs. −6.26 N·s·kg^−1^, *d*_s_ = 2.30) than the low-deceleration-ability group. Participants in the high-HDEC-ability group had a significantly higher approach velocity (7.80 vs. 7.37 m·s^−1^, *d*_s_ = 1.18) compared to the low-HDEC group, whereas participants in the high-HBI group had a significantly greater body mass (85.2 vs. 68.1 kg, *d*_s_ = 1.47), height (183 vs. 176 cm, *d*_s_ = 1.04) and approach momentum (651 vs. 507 kg·m·s^−1^, *d*_s_ = 1.83) than participants in the low-HBI group.

Table 3 reports comparisons between the high- and low-HDEC groups for all CMJ NMP variables. Only the concentric phase CMJ variables: peak force (25.87 vs. 23.53 N·kg^−1^, *p* = 0.02, *d*_s_ = 0.95), mean force (20.07 vs. 18.86 N·kg^−1^, *p* = 0.03, *d*_s_ = 0.91) and mean power (28.72 vs. 25.92 W·kg^−1^, *p* = 0.04, *d*_s_ = 0.85) were significantly higher in the high- compared to low-HDEC group. For the CMJ eccentric phase, the eccentric peak force (24.66 vs. 22.89 N·kg^−1^, *p* = 0.07, *d*_s_ = 0.72) was the only variable with a CL effect size ≥70% in the high- compared to low-HDEC group.

Table 4 shows comparisons between the high- and low-HBI groups for all CMJ NMP variables. Both CMJ concentric (2.76 vs. 2.52 m·s^−1^, *p* = 0.01, *d*_s_ = 1.15) and eccentric (−1.30 vs. −1.10 m·s^−1^, *p* = 0.02, *d*_s_ = −1.00) peak velocity had the largest phase-specific difference between the high- and low-HBI groups. Concentric peak power (−52.39 vs. 45.98 W·kg^−1^, *p* = 0.01, *d*_s_ = 1.06), mean power (28.76 vs. 25.67 W·kg^−1^, *p* = 0.02, *d*_s_ = 0.96) and impulse (2.62 vs. 2.38 N·s·kg^−1^, *p* = 0.01, *d*_s_ = 1.06) were also significantly higher in the high- compared to low-HBI group. The only other eccentric phase variables reporting a CL effect size ≥ 70% in the high- compared to low-HBI group was eccentric peak power (18.34 vs. 14.94 W·kg^−1^, *p* = 0.04, *d*_s_ = 0.86) and eccentric mean power (6.46 vs. 5.72 W·kg^−1^, *p* = 0.07, *d*_s_ = 0.73). CMJ height was also significantly higher (36.8 vs. 29.9 cm, *p* = 0.01, *d*_s_ = 1.07) in the high- compared to low-HBI group.

**Figure 3:**
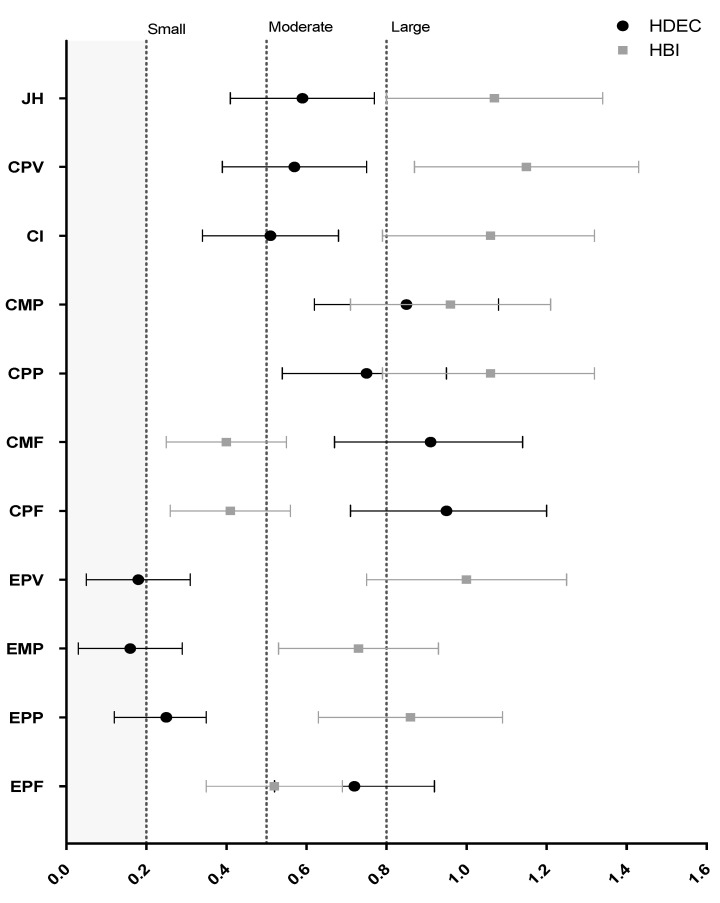
Comparison between the countermovement jump neuromuscular performance variables that best (CL effect size: ≥70%) differentiated athletes with “high” and “low” horizontal deceleration (HDEC, m·s^−2^) and horizontal braking impulse (HBI, N·s·kg^−1^). For simplicity, the eccentric peak velocity is shown as a positive effect size. The grey area represents a trivial effect size. JH—jump height, CPV—concentric peak velocity, CI—concentric impulse, CMP—concentric mean power, CPP—concentric peak power, CMF—concentric mean force, CPF—concentric peak force, EPV—eccentric peak velocity, EMP—eccentric mean power, EPP—eccentric peak power, EPF—eccentric peak force.

Figure 3 shows a comparison of the CMJ NMP variables that best (CL effect size: ≥70%) differentiated high and low HDEC and HBI. Only CMJ concentric mean and peak power had a CL effect size ≥70% for both HDEC and HBI. For the CMJ eccentric phase, only eccentric peak force had a moderate effect size difference for both HDEC (*d*_s_ = 0.72) and HBI (*d*_s_ = 0.52). For HDEC, both the CMJ eccentric (*d*_s_ = 0.72) and concentric (*d*_s_ = 0.52) peak forces represented the largest difference between the high- and low-deceleration-ability groups. However, for HBI, the largest difference was the eccentric (*d*_s_ = −1.00) and concentric (*d*_s_ = 1.15) peak velocities.

## 4. Discussion

The main finding of this study, in agreement with our hypothesis, was that specific CMJ concentric- and eccentric-phase NMP variables differentiated athletes with high versus low horizontal deceleration abilities. Interestingly, within both phases, the CMJ force variables had the largest effect size differences when using HDEC, while CMJ velocity variables best differentiated between high- and low-HBI athletes, with the latter being a deceleration measure that includes body mass in the calculation. These results suggest that heavier athletes, who perform horizontal decelerations with a higher approach momentum (high HBI = 651 kg m·s^−1^ vs. high HDEC = 566 kg m·s^−1^) may benefit from the development of different NMP qualities than the lower body mass (high HDEC = 73 kg vs. high HBI = 85 kg) high-HDEC-ability athletes.

In elite team sports competitive match-play, high-intensity decelerations are most commonly classified as a velocity change greater than −3 m·s^−2^ [2]. In the present study, when HDEC was used to define high and low horizontal deceleration ability, the average deceleration was −4.99 and −4.24 m·s^−2^, respectively. The CMJ NMP variables with the largest effect size difference between the high- and low-HDEC-ability groups were the concentric peak (*d*_s_ = 0.95) and the mean force (*d*_s_ = 0.91). While it may appear counterintuitive that concentric variables show greater significance to horizontal deceleration ability than eccentric variables, these findings agree with prior work in elite male youth soccer players demonstrating that concentric knee flexor and extensor peak torques at faster knee joint angular velocities were strongly correlated with horizontal deceleration ability [8].

There are a number of possible explanations regarding why the ability to produce concentric force at higher angular velocities may contribute to an improved HDEC ability. First, as suggested by Harper et al. [8] concentric muscle contractions can develop force more rapidly (RFD) than eccentric or isometric muscle actions, particularly at faster joint angular velocities [32]. Second, in both adolescent and senior athletes, the ability to produce high concentric forces at high joint angular velocities is associated with greater thickness and pennation angle of the leg extensor muscles, which are factors associated with greater isometric force, RFD and eccentric leg stiffness [33,34,35,36]. Increases in early (<100 ms) RFD, which are representative of explosive strength, become extremely important when high eccentric forces need to be produced when decelerating and could represent an ability to pre-activate muscles prior to ground contact [37]. Based on these findings interventions that enhance concentric force at fast joint angular velocities may provide important NMP adaptations that lead to an enhanced HDEC ability. Future research is needed to explore chronic adaptations to fast velocity resistance training and their transfer to horizontal deceleration abilities. Additionally, the ability to pre-activate muscles and produce high eccentric phase muscle activity augments the force output capabilities during the concentric phase [38]. As such, concentric outputs assessed during the CMJ are not independent of eccentric qualities and the ability to transfer force, and therefore represent important NMP characteristics underpinning a high HDEC ability.

Similar to the CMJ concentric phase variables, the eccentric peak force demonstrated the largest effect size difference (24.66 vs. 22.69 N·kg^−1^, *d*_s_ = 0.72) between the high- and low-HDEC-ability groups. The CL effect size was 70%, indicating that 7 times out of 10, an athlete with a high HDEC ability would also possess high eccentric peak force capabilities in the CMJ. Additionally, the high-HDEC-ability group showed a moderate effect size difference in eccentric-deceleration RFD (98.7 vs. 81.30 N·s^−1^·kg^−1^, *d*_s_ = 0.52). Collectively, these eccentric NMP qualities have also been associated with heightened leg stiffness [14,15,17,20,22], “stretch-load” tolerance [15], reactive strength [39] and the ability to rapidly unload the COM [14,15,24], which depends on rapid agonist relaxation and high lower limb joint flexion velocities [13]. Furthermore, a higher leg stiffness has also been associated with the ability to use a greater proportion of maximal isometric strength (calculated using the dynamic strength deficit (DSD)) [33], which is particularly important for enhancing eccentric-deceleration (braking) characteristics [40].

Previous studies have also found that higher eccentric peak torque capability in the knee extensors [3,8] and flexors [9] have strong associations with rapid horizontal deceleration abilities. However, to the present authors’ knowledge, no previous study has examined the associations between RFD and rapid horizontal deceleration ability. A significant increase in CMJ eccentric peak force was observed after only 5 weeks of ballistic jump squat (0% to 30% one repetition squat maximum-to-body-mass ratio (1RM/BM)) or heavy back squat training (75%–90% 1RM) in strong and weak athletes, respectively, with strength characterised by 3RM. Furthermore, these changes had a large and significant correlation (*r* = 0.92) with changes in CMJ total RFD [15]. Interestingly, in this study, the eccentric peak force (22.6 N·kg^−1^) values of the stronger athletes in the jump squat intervention were lower than those recorded in the current study (24.66 N·kg^−1^), but were higher following 5 and 10 weeks of jump squat training (27.6 and 30.6 N·kg^−1^, respectively). It is therefore possible that increases in maximal eccentric peak force have a significant influence on rapid eccentric force production capabilities (i.e., eccentric-deceleration RFD), which subsequently influence rapid HDEC ability.

On the basis of these findings and for the purpose of enhancing rapid HDEC ability, practitioners should identify training interventions targeting development of lower limb eccentric peak force and eccentric-deceleration RFD. For example, for the purposes of developing eccentric peak force, eccentric training methods that are not constrained by concentric strength levels, such as accentuated eccentric loading (AEL), have been shown to be superior than traditional resistance training methods [41,42]. Additionally, to target eccentric-deceleration RFD, training approaches that require the athlete to quickly decelerate the COM following a rapid acceleration should be utilised. In this context, the specificity of training would include fast eccentric only squats, where participants are instructed to “squat fast and stop rapidly at a half-squat position”, a modality shown in untrained subjects to induce greater adaptations to fast-twitch type IIX muscle fibres and explosive isometric RFD than slow eccentric-only squats [43]. Furthermore, these enhancements were also evident following low volume (4 × 8 reps, twice per week) interventions in moderately trained individuals [44].

Another key and novel finding of the current study was that when horizontal deceleration ability was defined using the change in momentum (HBI) instead of the HDEC method, the CMJ variables that best differentiated horizontal deceleration ability switched emphasis from force-based to velocity-based. For instance, the greatest differences between the high- and low-HBI groups were the CMJ concentric and eccentric peak velocities (2.72 vs. 2.52 m·s^−1^, *d*_s_ = 1.15; −1.30 vs. −1.10, *d*_s_ = 1.00) and peak power (52.39 vs. 45.98 W·kg^−1^, *d*_s_ = 1.06, and 18.34 vs. 14.94 W·kg^−1^, *d*_s_ = 0.86, respectively). A switch from a more force- to velocity-orientated power output has previously been associated with a CMJ strategy that utilises a greater countermovement depth (i.e., a more compliant strategy) [45,46]. Indeed, in the current study, the high-HBI athletes used a greater CMJ depth than the low-HBI athletes (−33.4 vs. −30.1 cm, respectively). Furthermore, the high-HBI athletes also produced significantly higher CMJ heights in comparison to the low-HBI athletes (36.8 vs. 29.9 cm, respectively). These findings agree with previous studies that have reported a deeper and faster countermovement to be crucial to jump performance outcomes, such as jump height, concentric peak velocity and impulse [46,47]. It is also possible that the greater CMJ depth and COM velocities deployed by the high-HBI group compared to the low-HBI group is indicative of a different deceleration strategy that is required to control higher forward momentum, and subsequently the greater deceleration demands [48]. Indeed, whilst there was only a small difference in the approach velocity (0.17 m·s^−1^) between the high- and low-HBI groups, the high-HBI group was on average 18 kg heavier than the low-HBI group, resulting in a 22% greater approach momentum prior to decelerating. In accordance with the findings of Cesar and Sigward [48,49] it is therefore possible that the high-HBI athletes adopted a lower (knee-dominant) and more posterior COM position compared to the low-HBI peers to maintain stability and decelerate effectively, which is reflective in their CMJ movement strategy and NMP qualities. Therefore, to enhance the ability to quickly reduce their momentum (i.e., high HBI), these findings suggest that training interventions should focus upon maximising the mechanical power output through fast velocity eccentric-to-concentric movement actions. Furthermore, whilst it is common practice to evaluate the effect of training on concentric peak power, only a few studies have investigated training-induced changes in eccentric peak power [15,16]. Since the findings of this study identify the potential importance of eccentric peak power for HBI performance, future studies should evaluate this component in other athletic populations and determine whether improvements in eccentric peak power correlate with improvements in HBI.

Based on a large number of studies reporting large correlations between maximal strength and external mechanical power output, the development of maximal strength is considered to be the foundation upon which external mechanical power output is built [50]. This suggests (as discussed previously) that the development of maximal strength should be prioritised in the early phases of a training program aimed at developing rapid HBI performance. Consequently, it is possible that the high-HBI athletes had a higher maximal strength potential than the low-HBI athletes, enabling them to generate higher velocities, and in turn, greater eccentric and concentric power. However, it should be highlighted that despite stronger (squat 1RM/BM) and more powerful (jump squat peak power) athletes having superior sprint acceleration momentum and jumping ability, they may not also be superior in movements that involve a significant horizontal deceleration component, such as when performing a more severe COD task [51,52]. This apparent discrepancy may be associated with an over-emphasis on the development of horizontal sprint acceleration performance, in tandem with an under-emphasis on the development of the technical skills and mechanical capabilities underpinning horizontal deceleration ability. Given the demonstrated frequency and intensities of decelerations [2] and deceleration-dependent COD activities during team sports match play, addressing this imbalance has potential performance and injury resilience benefits to players.

Indeed evidence is accumulating showing that strength training interventions that incorporate and effectively manipulate AEL could enhance HBI and COD ability [53]. Accordingly, in a recent review, AEL and plyometric training was considered as having the best theoretical potential for enhancing mechanical power output [42]. Subsequently, recommendations on how best to implement these have also been suggested [54]. Additionally, other eccentric exercise modalities, such as flywheel inertial resistance training [55], that were not included in this review have also been shown to be effective in enhancing AEL and mechanical power output, and should therefore also be considered in a training schedule that is focused upon developing HBI performance.

A limitation of the current study was the cross-sectional research design, thus conclusions cannot be made on whether the NMP characteristics found to differentiate between high and low horizontal deceleration abilities will actually transfer to enhanced horizontal deceleration ability following a long-term training period. Therefore, future long-term training studies should evaluate this potential transfer. Furthermore, the current study used a sample of young male university athletes, and therefore, the NMP qualities identified to be most important for horizontal deceleration may not be generalizable to female athletes, or to male and female athletes with different performance characteristics, levels and sports. Future research should examine these associations across and within other populations. Finally, given that the CMJ test was performed both bilaterally and in the vertical plane, it would also be useful to examine performance and kinetic variables in jump tests with either (or both of) a unilateral and horizontal braking GRF component. NMP tests with a progressively greater eccentric demand, such as loaded jumps and drop jumps, may show greater importance for horizontal deceleration abilities and should be evaluated in future research. Additionally, in this study, eccentric-deceleration RFD was averaged across the entire eccentric-deceleration phase, as previously described [16,17]. Given the potential importance of this metric as an indicator of horizontal deceleration ability, the evaluation of time-constrained eccentric-deceleration RFD should also be examined in future investigations, such as the first 50 to 100 ms [14] or as a percentage of the phase.

## 5. Conclusions

This study aimed to determine whether NMP qualities determined using the CMJ could differentiate team sport athletes characterised with high or low horizontal deceleration abilities. Importantly, greater eccentric and concentric peak velocities differentiated athletes with high change in momentum abilities, defined as horizontal braking impulse, whereas eccentric and concentric peak force differentiated athletes with high average deceleration abilities. The analysis notably highlighted the importance of quantifying the change in momentum ability, particularly for heavier athletes, when evaluating horizontal deceleration ability.

Essentially, these results demonstrated a switch in emphasis from force-based to velocity-based power production when athletes were categorised using HBI in comparison to the HDEC approach, which could be indicative of different horizontal deceleration strategies. Subsequently, when measuring a player’s maximal horizontal deceleration ability, we recommend that practitioners should consider both the HDEC and HBI performances. These are derived from the same test and do not require additional data collection, and both may inform decisions based on the individual’s deceleration profile relative to group-based normative data. For the purposes of obtaining an indirect neuromuscular indicator of a player’s horizontal deceleration capacity, concentric mean power was the only variable that differentiated both higher and lower performers characterized with both HDEC and HBI. Accordingly, based on currently available evidence, this metric would seem to be the best overall indicator.

Despite the significance of the eccentric phase to horizontal deceleration ability, our findings demonstrate that concentric phase variables had the largest differences between both high and low horizontal deceleration abilities (HDEC and HBI). Subsequently, these findings suggest concentric force and velocity should also be considered important NMP determinants of horizontal deceleration ability. These findings, accordingly, have important implications for coaches and sport science professionals tasked with preparing team sport athletes for competition demands. Given the significance of maximal horizontal deceleration ability to team sport performance [2], injury risk [6] and the effectiveness of return-to-play protocols [56], future research should (a) investigate the NMP determinants of horizontal deceleration ability across different sports and performance levels, and (b) seek to determine the efficacy of training interventions specifically focused on improving maximal horizontal deceleration ability.

## Figures and Tables

**Table 1:** Definitions of countermovement jump neuromuscular performance variables and absolute reliability values.

CMJ Variables	Description	CV%
**Concentric**		
Peak Force (N·kg^−1^)	Greatest force achieved during the concentric phase	2.7
Mean Force (N·kg^−1^)	Mean force during the concentric phase	1.5
Peak Power (W·kg^−1^)	Greatest power achieved during the concentric phase	1.8
Mean Power (W·kg^−1^)	Mean power during the concentric phase	2.1
Impulse (N·s·kg^−1^)	Concentric force exerted multiplied by the time taken	1.4
Peak Velocity (m·s^−1^)	Greatest velocity achieved during the concentric phase	1.1
Duration (ms)	Duration of the concentric phase	2.6
**Eccentric**		
Peak Force (N·kg^−1^)	Greatest force achieved during the eccentric phase	3.0
Peak Power (W·kg^−1^)	Greatest power during the eccentric phase from the start of the movement to zero velocity	8.8
Mean Power (W·kg^−1^)	Mean power during the eccentric phase from the start of the movement to zero velocity	4.7
Peak Velocity (m·s^−1^)	Greatest velocity achieved during the eccentric phase	5.6
Duration (ms)	Time from the start of the movement to zero velocity	3.4
**Eccentric Deceleration**		
Mean Force (N·kg^−1^)	Mean force from the greatest negative velocity to zero velocity at the end of the eccentric phase	3.2
Impulse (N·s·kg^−1^)	Force exerted multiplied by the time taken from the greatest negative velocity to zero velocity at the end of the eccentric phase	3.0
RFD (N·s^−1^·kg^−1^)	Rate of force development from the greatest negative velocity to zero velocity at the end of the eccentric phase	9.3
Duration (ms)	Time from the maximum negative velocity to zero velocity at the end of the eccentric phase	4.1
**Other**		
CMJ-Height (cm)	Maximal jump height computed using the flight time	2.9
CMJ-Depth (cm)	Maximal displacement of countermovement	2.9
RSI-Mod	Jump height (calculated from flight time) divided by contraction time	4.3

CV%—Coefficient of Variation Percentage, CMJ—Countermovement Jump, RFD—Rate of Force Development, RSI—Reactive Strength Index.

**Table 2:** Descriptive information showing the differences between the high- and low-horizontal-deceleration (HDEC) and -horizontal-braking-impulse (HBI) groups.

Variable	High HDEC (n = 13)	Low HDEC (n = 14)	ES (*d*_s_)	High HBI (n = 14)	Low HBI (n = 13)	ES (*d*_s_)
Age (y)	19.7 ± 1.6	20.4 ± 2.3	0.35	20.1 ± 2.2	19.1 ± 1.1	0.57
Body Mass (kg)	72.7 ± 16.0	80.7 ± 12.4	0.56	85.2 ± 12.5	68.1 ± 10.6	1.47 **
Height (cm)	180 ± 9	180 ± 8	0.00	183 ± 8	176 ± 5	1.04 **
Approach Velocity (m·s^−1^)	7.80 ± 0.44	7.37 ± 0.28	1.18 **	7.66 ± 0.45	7.49 ± 0.38	0.41
Approach Momentum (kg·m·s^−1^)	566 ± 123	594 ± 94	0.26	651 ± 90	507 ± 64	1.83 **
HDEC (m·s^−2^)	−4.99 ± 0.24	−4.24 ± 0.41	2.21 **	−4.72 ± 0.39	−4.48 ± 0.59	0.48
HBI (N·s·kg^−1^)	−7.56 ± 1.66	−7.22 ± 1.24	0.23	−8.43 ± 1.15	−6.26 ± 0.65	2.30 **

ES—Effect Size (Cohen’s *d*_s_); ** p ≤ 0.01.

**Table 3:** Countermovement jump (CMJ) neuromuscular performance qualities that differentiate between athletes with a high and low horizontal deceleration (HDEC).

Variable	High HDEC (n = 13)	Low HDEC (n = 14)	ES (90% CI)	CL-ES	Descriptor	*p*-Value
**Concentric**						
Peak Force (N·kg^−1^)	25.87 ± 2.42	23.53 ± 2.50	0.95 (0.71, 1.20)	75%	Large	0.02 *
Mean Force (N·kg^−1^)	20.07 ± 1.27	18.86 ± 1.39	0.91 (0.67, 1.14)	74%	Large	0.03 *
Peak Power (W·kg^−1^)	51.81 ± 7.17	46.98 ± 5.68	0.75 (0.54, 0.95)	70%	Moderate	0.06
Mean Power (W·kg^−1^)	28.72 ± 2.84	25.92 ± 3.66	0.85 (0.62, 1.08)	73%	Large	0.04 *
Impulse (N·s·kg^−1^)	2.57 ± 0.27	2.44 ± 0.24	0.51 (0.34, 0.68)	64%	Moderate	0.20
Peak Velocity (m·s^−1^)	2.71 ± 0.25	2.58 ± 0.21	0.57 (0.39, 0.75)	65%	Moderate	0.15
Duration (ms)	249 ± 39	271 ± 39	−0.56 (−0.39, −0.73)	66%	Moderate	0.16
**Eccentric**						
Peak Force (N·kg^−1^)	24.66 ± 2.42	22.89 ± 2.47	0.72 (0.52, 0.92)	70%	Moderate	0.07
Peak Power (W·kg^−1^)	17.47 ± 3.82	16.38 ± 4.86	0.25 (0.12, 0.38)	57%	Small	0.53
Mean Power (W·kg^−1^)	6.35 ± 1.10	6.17 ± 1.16	0.16 (0.03, 0.29)	54%	Trivial	0.68
Peak Velocity (m·s^−1^)	−1.22 ± −0.21	−1.18 ± 0.24	−0.18 (−0.31, −0.05)	55%	Trivial	0.65
Duration (ms)	485 ± 58	514 ± 88	−0.39 (−0.24, −0.54)	61%	Small	0.33
**Eccentric Deceleration**						
Mean Force (N·kg^−1^)	18.10 ± 1.41	17.30 ± 2.10	0.44 (0.28, 0.60)	62%	Small	0.26
Impulse (N·s·kg^−1^)	2.88 ± 0.48	2.90 ± 0.44	−0.04 (−0.16, 0.08)	51%	Trivial	0.91
RFD (N·s^−1^·kg^−1^)	98.7 ± 34.4	81.3 ± 25.4	0.58 (0.40 to 0.76)	66%	Moderate	0.15
Duration (ms)	160 ± 30	170 ± 30	−0.33 (−0.19, −0.47)	59%	Small	0.40
**Other**						
CMJ Height (cm)	35.7 ± 7.8	31.5 ± 6.3	0.59 (0.41, 0.77)	66%	Moderate	0.14
CMJ Depth (cm)	31.7 ± 7.9	32.4 ± 6.7	0.11 (−0.02, 0.23)	53%	Trivial	0.94
RSI-Mod (m·s^−1^)	0.45 ± 0.11	0.42 ± 0.09	0.27 (0.13, 0.40)	58%	Small	0.44

ES—Effect Size (Cohen’s *d*_s_); CL—Common Language; CI—Confidence Interval; RFD—Rate of Force Development; RSI-Mod—Reactive Strength Index Modified. * p < 0.05.

**Table 4:** Countermovement jump (CMJ) neuromuscular performance qualities that differentiate between athletes with a high and low horizontal braking impulse (HBI).

Variable	High HBI (n = 14)	Low HBI (n = 13)	ES (90% CI)	CL-ES	Descriptor	*p*-Value
**Concentric**						
Peak Force (N·kg^−1^)	25.19 ± 2.56	24.09 ± 2.82	0.41 (0.26, 0.56)	61%	Small	0.30
Mean Force (N·kg^−1^)	19.72 ± 1.15	19.14 ± 1.71	0.40 (0.25, 0.55)	61%	Small	0.31
Peak Power (W·kg^−1^)	52.39 ± 7.12	45.98 ± 4.63	1.06 (0.79, 1.32)	77%	Large	0.01 *
Mean Power (W·kg^−1^)	28.76 ± 3.67	25.67 ± 2.65	0.96 (0.71, 1.21)	75%	Large	0.02 *
Impulse (N·s·kg^−1^)	2.62 ± 0.28	2.38 ± 0.15	1.06 (0.79, 1.32)	78%	Large	0.01 *
Peak Velocity (m·s^−1^)	2.76 ± 0.25	2.52 ± 0.15	1.15 (0.87, 1.43)	79%	Large	0.01 **
Duration (ms)	262 ± 27	259 ± 51	0.07 (−0.05, 0.19)	52%	Trivial	0.85
**Eccentric**						
Peak Force (N·kg^−1^)	24.37 ± 2.71	23.07 ± 2.30	0.52 (0.35, 0.69)	64%	Moderate	0.19
Peak Power (W·kg^−1^)	18.34 ± 3.45	14.94 ± 4.46	0.86 (0.63, 1.09)	73%	Large	0.04 *
Mean Power (W·kg^−1^)	6.46 ± 0.68	5.72 ± 1.27	0.73 (0.53, 0.93)	70%	Moderate	0.07
Peak Velocity (m·s^−1^)	−1.30 ± 0.14	−1.10 ± 0.25	−1.00 (−0.75, −1.25)	76%	Large	0.02 *
Duration (ms)	493 ± 55	507 ± 95	−0.18 (−0.05, −0.31)	55%	Trivial	0.64
**Eccentric Deceleration**						
Mean Force (N·kg^−1^)	18.25 ± 1.96	17.08 ± 1.48	0.67 (0.48 to 0.86)	68%	Moderate	0.09
Impulse (N·s·kg^−1^)	2.99 ± 0.30	2.79 ± 0.56	0.45 (0.29 to 0.61)	62%	Small	0.25
RFD (N·s^−1^·kg^−1^)	93.6 ± 34.4	85.6 ± 27.2	0.26 (0.12 to 0.39)	57%	Small	0.51
Duration (ms)	160 ± 20	160 ± 30	0.00 (−0.12 to 0.12)	50%	Trivial	1.00
**Other**						
CMJ Height (cm)	36.8 ± 7.8	29.9 ± 4.5	1.07 (0.80, 1.34)	78%	Large	0.01 *
CMJ Depth (cm)	−33.4 ± 5.7	−30.1 ± 8.2	−0.47 (−0.31, −0.63)	63%	Small	0.23
RSI-Mod (m·s^−1^)	0.43 ± 0.09	0.43 ± 0.11	0.00 (−0.12, 0.12)	50%	Trivial	1.00

ES—Effect Size (Cohen’s *d*_s_); CL-ES—Common Language; CI—Confidence Interval; RFD—Rate of Force Development; RSI-Mod—Reactive Strength Index Modified. * *p* < 0.05, ** *p* < 0.01.
